# Optical Constants of Crystallized TiO_2_ Coatings Prepared by Sol-Gel Process

**DOI:** 10.3390/ma6072819

**Published:** 2013-07-12

**Authors:** Xiaodong Wang, Guangming Wu, Bin Zhou, Jun Shen

**Affiliations:** Shanghai Key Laboratory of Special Artificial Microstructure Materials and Technology, Pohl Institute of Solid State Physics, Tongji University, Siping Road 1239, Shanghai 200092, China; E-Mails: wugm@tongji.edu.cn (G.W.); zhoubin863@tongji.edu.cn (B.Z.)

**Keywords:** sol-gel, TiO_2_ coating, thermal annealing, optical constants

## Abstract

Titanium oxide coatings have been deposited by the sol-gel dip-coating method. Crystallization of titanium oxide coatings was then achieved through thermal annealing at temperatures above 400 °C. The structural properties and surface morphology of the crystallized coatings were studied by micro-Raman spectroscopy and atomic force microscopy, respectively. Characterization technique, based on least-square fitting to the measured reflectance and transmittance spectra, is used to determine the refractive indices of the crystallized TiO_2_ coatings. The stability of the synthesized sol was also investigated by dynamic light scattering particle size analyzer. The influence of the thermal annealing on the optical properties was then discussed. The increase in refractive index with high temperature thermal annealing process was observed, obtaining refractive index values from 1.98 to 2.57 at He-Ne laser wavelength of 633 nm. The Raman spectroscopy and atomic force microscopy studies indicate that the index variation is due to the changes in crystalline phase, density, and morphology during thermal annealing.

## 1. Introduction

Titanium oxide (TiO_2_) coatings have been extensively studied and used owing to their great potential in optical device application, such as planar waveguides [1], antireflective coatings [2,3], and solar cells [4,5,6]. Various methods are available for the preparation of the TiO_2_ coatings, such as electron-beam evaporation [7,8], sputtering [9,10], pulsed laser deposition [11,12,13], chemical spray pyrolysis [14], chemical vapor deposition [15,16], and the sol-gel method [17,18,19,20,21]. Among the different techniques, the sol-gel method seems to be the most promising one as it offers the advantages of macro-porous coatings prepared at a comparatively low cost, capability of large coating size, and relatively easy process control [22].

As sol-gel method is a type of wet chemical method, the as-deposited coating is amorphous. Thermal annealing can be used for crystallization of TiO_2_ coatings, which will endow the TiO_2_ coating with higher refractive index [23], self-cleaning property [24], and photo-catalytic activity [25]. For sol-gel derived TiO_2_ coating, the high porosity combined with the existence of residual organic components make the optical constants of coating layer quite different from that of solid coating and bulk materials. Therefore, it is very important to determine the optical constants for sol-gel derived coatings systemically. In our previous work [26], the optical constants of amorphous TiO_2_ coatings were determined by spectroscopic ellipsometry, and these amorphous TiO_2_ coatings have been successfully used for fabrication of two-layer antireflective coatings [3]. However, the reports available for optical constants of crystallized sol-gel TiO_2_ coatings are scarce and not yet taken seriously. It is of interest to study the optical constants of the crystallized sol-gel TiO_2_ coatings. Hence, we performed this work to understand the optical properties of crystallized sol-gel TiO_2_ coatings more systemically and deeply. The TiO_2_ coatings are prepared by sol-gel dip-coating method and annealed between 400 and 900 °C. The variation of the refractive index of the coatings was measured through spectrometer and optical model fitting. Raman spectroscopy was conducted to explore the crystalline nature of the coating. The atomic force microscope (AFM) was used to measure the surface morphology. The crystalline structure, surface morphology, and optical properties of the TiO_2_ coatings were then studied and discussed. We demonstrated that the increase in refractive index was due to combined effects of crystalline phase, density, and morphology during thermal annealing.

## 2. Experimental Section

### 2.1. TiO_2_ Sol Synthesis

**Figure 1:**
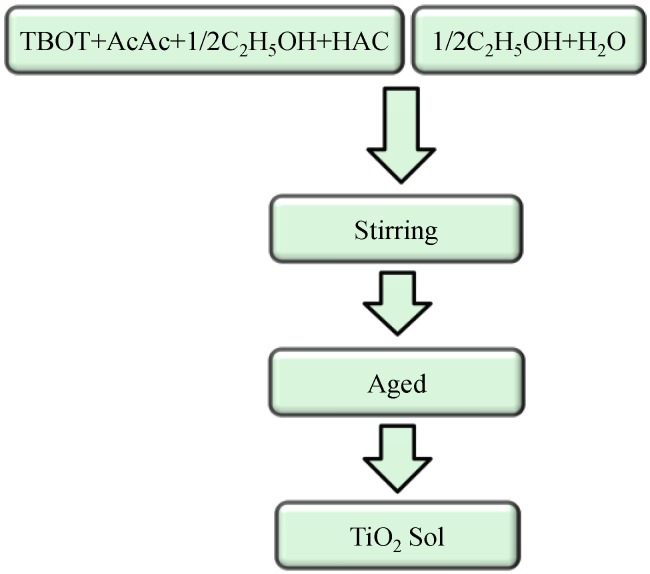
Flow chart of TiO_2_ sol synthesis process.

The flow chart of the TiO_2_ sol synthesis process is shown in Figure 1. Tetrabutyl titanate (Ti(OC_4_H_9_)_4_, TBOT) was selected as precursor, with anhydrous ethanol (C_2_H_5_OH, EtOH) as solvent, deionized water for hydrolysis, acetylacetone (CH_3_COCH_2_COCH_3_, AcAc) as chelating agent and acetic acid (CH_3_COOH, HAc) as catalyst. During synthesis, two different but equal parts of ethanol solutions were prepared. In the first part, TBOT was dissolved into anhydrous ethanol containing AcAc. After mixing with HAc, the solution was then sealed and kept stirring for 30 min to achieve a complete chelation between the alkoxide and AcAc. The second part of the solution was then prepared by mixing the deionized water with anhydrous ethanol. These two solutions were then mixed and stirred for 2 h to achieve hydrolysis and condensation. The molar ratio was TBOT:EtOH:H_2_O:HAc:AcAc = 1:30:3:2:1. The mixture was finally aged in a stable environment (with humidity lower than 30% and temperature of 20~25 °C) for 72 h.

### 2.2. Coating Preparation

The silicon wafer and silica glass substrates were firstly cleaned thoroughly, heated at 200 °C for 20 min, and then cooled down to room temperature. A dip-coating apparatus (CHEMAT Dip Master-200) was used for the depositions, and the coating thickness could be adjusted by the withdrawal rate (0~12 inch/min). After each coating, the coatings were first pretreated at 100 °C for 1 h, and then heat-treated in a muffle furnace for 2 h at different temperatures, ranging from 300 to 900 °C. All the coating processes of the samples were the same to make sure the properties of coatings annealed at different temperatures can be compared and studied.

### 2.3. Characterization

Particle size distribution of TiO_2_ sol was analyzed using a dynamic light scattering particle size analyzer (HORIBA LB-550). Raman spectra were recorded at room temperature with a JOBIN-YVON micro-Raman apparatus (HR-800) equipped with a 30 mW He-Cd laser (KIMMON KOHA, IK3301R-G) emitting at 325 nm, and a microscope (OLYMPUS BX41). An edge filter was used in the Raman setup to block the Rayleigh scattering light and stray laser bands. The laser beam irradiating the sample was attenuated to below 0.1 mW in order to avoid laser-induced heating. Before each measurement, a standard silicon wafer was used for the wavenumber calibration of the spectrometer. The spectral resolution was estimated to be 1.6 cm^−1^. The transmittance and reflectance spectra of the coatings were measured in the 400~1000 nm region using a JASCO-V570 UV-VIS-NIR double beam spectrometer. The surface morphology of the coatings was characterized using AFM (PSIA XE-100).

## 3. Results and Discussion

### 3.1. Structural Properties

**Figure 2:**
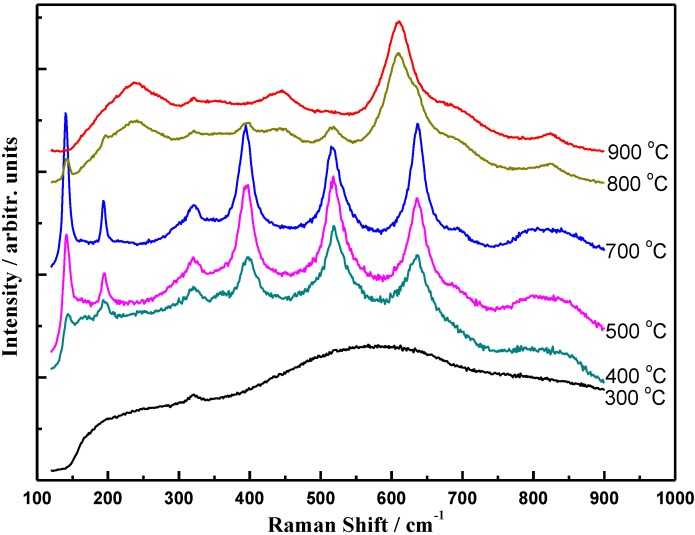
Raman spectra of TiO_2_ coatings annealed at different temperatures.

Figure 2 shows the micro-Raman spectra of the TiO_2_ coatings annealed at different temperatures. It can be seen from the figure that crystallization does not start until the annealing temperature reaches 400 °C. Raman spectra of the coatings annealed at temperatures higher than 400 °C exhibit clearly lines characteristic of anatase TiO_2_ phase at 141, 194, 394, 515, and 636 cm^−1^. The intensity of these lines increases with increasing annealing temperature. For temperature up to 800 °C, besides the peaks characteristic of the anatase phase, the new Raman peaks at 144, 235, 443, 610, and 824 cm^−1^ that come from the rutile TiO_2_ are also observed, which means that the coatings were composed of anatase and rutile structures at this temperature. The observed band positions are in complete accordance with those reported in previous studies for anatase and rutile powder and single crystals [22,27,28]. At 900 °C, the peaks from anatase TiO_2_ disappear, indicating the completion of the transformation of TiO_2_ coatings from anatase to rutile phase.

### 3.2. Optical Properties

**Figure 3:**
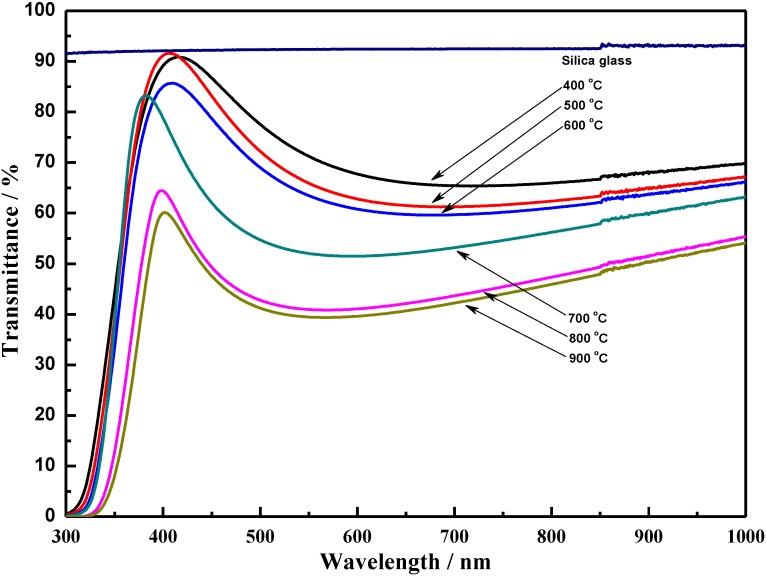
Transmittance spectra of TiO_2_ coatings annealed at different temperatures.

Transmittance spectra of the coatings annealed at various temperatures in the wavelength region from 300 to 1000 nm are presented in Figure 3. Silica glasses were used as the substrate in these experiments to avoid the influence of the absorption edge of the substrate. As shown in Figure 3, the average transmittance of 400 °C annealed TiO_2_ coatings is about 80% in the visible region with respect to silica glass substrate. Annealing shows a slight decrease in transmittance with the increasing annealing temperature. The coatings annealed above 700 °C shows a significant decrease in visible light transmittance. It can be attributed to the densification and the crystalline transformation that increase the refractive index of the TiO_2_ coatings. Then the refractive index has been determined to see the direct effect of densification and crystalline structure.

The determination of the refractive index of the coatings can be inferred from the spectroscopic measurements. For sol-gel dip-coating technology, both sides of the glass substrates were coated. Therefore, we built a five layer (Air/TiO_2_ layer/Silica glass/TiO_2_ layer/Air) structure model to determine the optical constants of the coating. The Cauchy formula [29], which is an optical model for insulators and dielectric coatings, was used to describe the dispersion relationship of the coating layer.

(1) $$n(\lambda )=A_{n}+10^{6}B_{n}/\lambda^{2}+10^{12}C_{n}/\lambda^{4},\text{where }\lambda (\text{Wavelength})\text{ in nm}$$

(2) $$k(\lambda )=A_{k}+10^{6}B_{k}/\lambda^{2}+10^{12}C_{k}/\lambda^{4},\text{where }\lambda (\text{Wavelength})\text{ in nm}$$

The optical parameters of the TiO_2_ layer were then determined from the measured transmittance and reflectance spectra for each coating by fitting simultaneously the results obtained from calculations and measurements over the 400~1000 nm region. The detailed fitting process can be founded in our previous work [30,31]. To judge the quality of the fitting, the root mean squared error (*RMSE*) was defined by:

(3) $$RMSE\text{=}\sqrt{\sum_{j=1}^{n}\left[{\left(Y_{\exp_{j}}-Y_{calc_{j}}\right)}^{2}\times weight_{j}^{2}\right]/\sum_{j=1}^{n}weight_{j}^{2}},$$

where *n* is the number of selected experimental data, *Y_exp_* is the value of the experimental data, *Y_calc_* is the calculated value and *weight_j_* is the weight of each experimental point. The lower the RMSE value, the better is the agreement between fit and experimental data. The calculated optical parameters of all the coatings are listed in Table 1. All the *RMSE* values are less than 1, which ensure the reliability of the determined optical constants.

**Table 1:** Caculated optical parameters of TiO_2_ coatings.

Sample	*A_n_*	*B_n_* (nm)	*C_n_* (nm)	*A_k_*	*B_k_* (nm)	*C_k_* (nm)	Thickness *d* (nm)	*RMSE*
400 °C	1.892	0.036	5.701 × 10^−4^	3.508 × 10^−3^	1.538 × 10^−15^	2.567 × 10^−10^	96.46	0.4452
500 °C	1.958	0.040	1.704 × 10^−3^	5.757 × 10^−3^	2.629 × 10^−13^	7.540 × 10^−13^	89.94	0.6306
600 °C	1.996	0.035	3.921 × 10^−15^	7.965 × 10^−3^	2.280 × 10^−9 ^	1.259 × 10^−9^	86.87	0.5689
700 °C	2.127	0.051	1.255 × 10^−3^	8.741 × 10^−3^	2.054 × 10^−12^	4.287 × 10^−9^	70.70	0.4936
800 °C	2.401	0.023	0.010	2.987 × 10^−3^	4.708 × 10^−3^	4.310 × 10^−4^	61.81	0.8760
900 °C	2.385	0.063	4.707 × 10^−3^	0.011	5.985 × 10^−11^	1.102 × 10^−3^	61.02	0.3872

**Figure 4:**
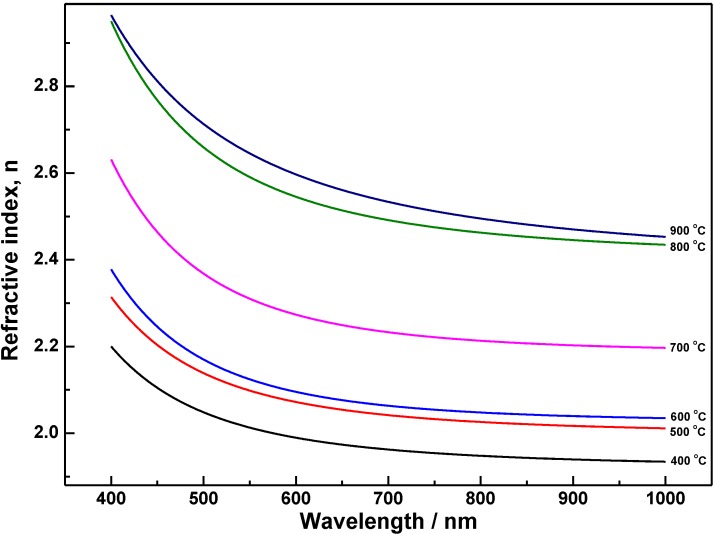
Refractive index dispersion of TiO_2_ coatings annealed at different temperatures.

The determined refractive indices, as a function of wavelength for crystallized TiO_2_ coatings annealed at different temperatures, are shown in Figure 4. Decreasing refractive index with wavelength indicates normal dispersion behavior. The data show that the refractive index increases with annealing temperature, from 1.98 to 2.57 at He-Ne laser wavelength of 633 nm. The highest refractive index of anatase TiO_2_ coating reaches to 2.26, which was close to the bulk anatase TiO_2_. At 700 °C annealed coating, the rapid increase of refractive index might due to the thermal induced growth of the grains which increased the packing density. Further increasing annealing temperatures promotes the transition of crystalline phase from anatase to rutile and the densification of the coating structures [32]. Therefore, the refractive index has another rapid increase at 800 °C annealed coating. As can be known from AFM analysis, the crystalline transformation not only increases the density of the coatings but also leaves pinholes, which will decrease the packing density of the coating. Thus, the refractive index of rutile TiO_2_ coating (900 °C annealed) only has a slight increase compared with the mixed phase TiO_2_ coating (800 °C annealed).

Also, it is noticeable from Figure 3 that all the coatings are transparent in the visible light region and their spectra exhibit a sharp decrease in the UV region (300~350 nm) because of the fundamental light absorption. From the measured transmittance *T*, given in Figure 3, we deduce the absorption coefficient *α* using the following relation [33]:

(4) $$\alpha \approx \frac{1}{d}\ln\frac{1}{T}$$

where *d* is the thickness of the coating (Table 1) and *T* is its transmittance.

**Figure 5:**
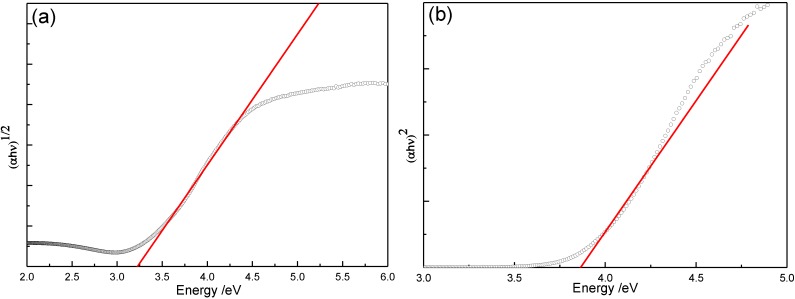
Plot of (*αhν*)^1/m^ *vs*. (*hν*) for the estimation of the band gap energy value (400 °C annealed TiO_2_ coating): (**a**) indirect gap and (**b**) direct gap.

It is known that TiO_2_ has direct and indirect band gaps [34]. To determine values of these forbidden energies, we use the expression in Equation (5). The relationship between the absorption coefficient *α* and the incident photon energy is given by the relation as follows [35]:

(5) $$\alpha h\nu =A（h\nu -E_{g}{）}^{m}$$

where *A* is a constant depending on the transition probability and *m* is equal to 1/2 for a direct gap and 2 for an indirect gap. The usual method to calculate the band gap energies is to plot (*αhν*)^1/^*^m^* as a function of the incident radiation energy (*hν*) [36]. The band gap values are then determined by extrapolating values of the absorption coefficient *α* to zero. Figure 5 is a typical plot of (*αhν*)^1/^*^m^* *vs*. incident energy (*hν*). As represented in the figure, we found 3.86 and 3.22 eV for the direct and indirect band gap energies, respectively.

The same analysis procedure was repeated to obtain the band gaps for all the TiO_2_ coatings annealed at different temperatures (as listed in Table 2). As can be seen, the direct band gap decreased from 3.86 to 3.65 eV with increasing of annealing temperature from 400 to 900 °C, while the indirect band gap decreased from 3.22 to 2.93 eV. It is connected with the size of the grain. An increase of the grain size weakens quantum size effects, thereby causing the decrease of the band gap and red shift of the absorption edge.

**Table 2:** Band gap of TiO_2_ coatings annealed at different temperatures.

Sample/°C	400	500	600	700	800	900
*E_g-indirect_*/eV	3.22	3.17	3.15	3.09	2.97	2.93
*E_g-direct_*/eV	3.86	3.82	3.79	3.77	3.67	3.65

### 3.3. Surface Morphology

**Figure 6:**
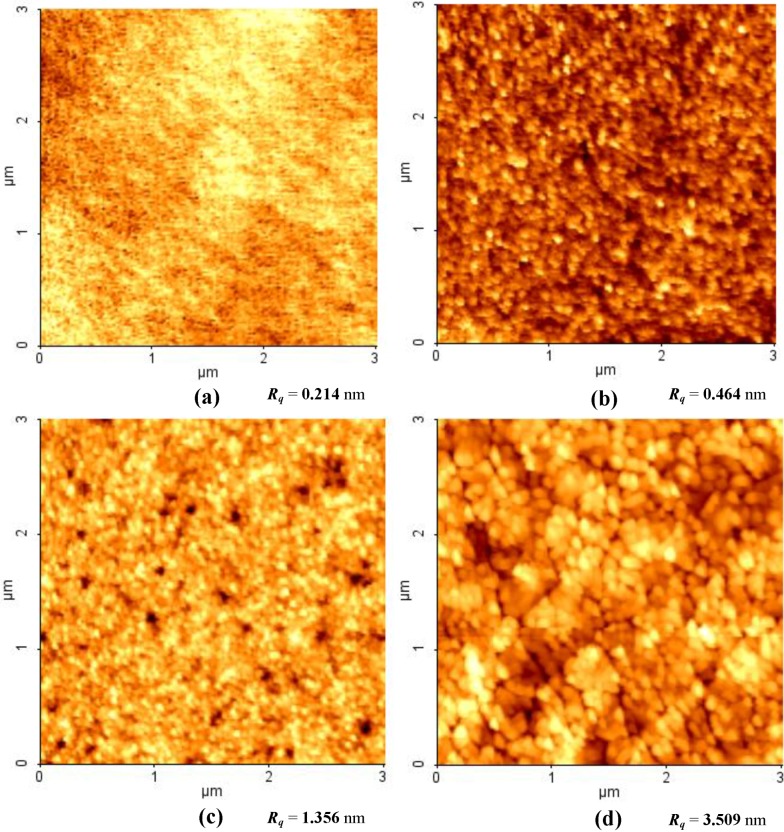
2D AFM images of TiO_2_ coatings annealed at (**a**) 300 °C; (**b**) 500 °C; (**c**) 700 °C; (**d**) 900 °C.

The AFM imaging was performed to study the surface morphology changes induced by thermal annealing. Figure 6 shows the surface morphology of the coatings annealed at 300, 500, 700, and 900 °C, respectively. Three hundred-degree Celsius annealed coating exhibits a smooth surface and very fine particles, indicating the amorphous structure of the coating. The root mean square roughness (*R_q_*) of the surface is less than 1 nm and increases with the increasing treatment temperature, which indicates the grains of regular shapes develop on the surface.

As can be seen from the Figure 6b (coating treated at 500 °C), there is a preferred orientation of the regularly-shaped grains, which suggests the growth of the nano-crystalline TiO_2_ grains. At temperatures of 700 °C, the grains get larger and combine to make denser coatings, but the basic structure remains un-changed. After annealing up to 900 °C, a significant increase of the grain size is observed and *R_q_* increases to 3.509 nm. It is because the transformation of anatase into rutile went through the coalescence of smallest particles and formation of bigger particles [37], which then increase its grain size. The AFM results are in good agreement with Raman analysis, which shows structure changes during thermal annealing. Coating densification takes place with coating crystallization, as also revealed by AFM.

### 3.4. Stability of TiO_2_ Sol

**Figure 7:**
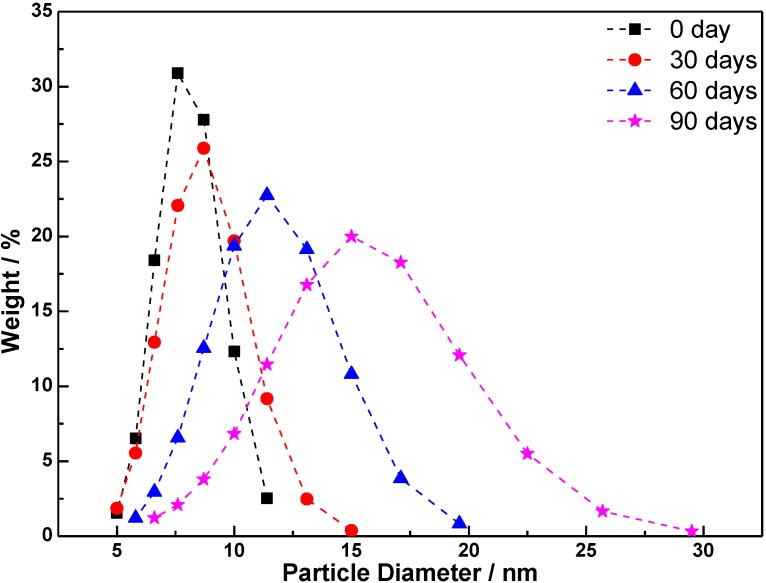
Particle size distribution of TiO_2_ sol.

The sol stability plays an important role in obtaining the uniform TiO_2_ coatings with a high optical quality, especially for industrial application. During the storage, the particles and clusters keep growing and the network keeps extending until the sol finally turns to a gel, which will affect the properties of the final products. Particle size distribution curve of as-synthesized sol, presented in Figure 7, shows a narrow distribution of titania particles with an average size of 7.4 nm. The measurement was repeated every 30 days to evaluate the stability in the long term storage at the room temperature. As the figure showed, the average size of the TiO_2_ particles after 30 and 60 days are 8 and 10.6 nm, respectively. After being stored for 90 days, they increased to 14 nm, which is still in a sufficient condition for coating. This kind of gradual evolution can be attributed partly to the esterification reaction, which allows slow continuing further hydrolysis to occur and helps combine the particles with time [38,39]. This indicates that the reaction during the sol-gel process was controlled with a relatively optimized condition and can be used for coating in a relatively long time.

## 4. Conclusions

TiO_2_ coatings have been deposited by the sol-gel method and the coatings were annealed at temperatures from 400 to 900 °C to realize crystallization. The Raman analysis show that the 400 °C annealed TiO_2_ coatings are crystallized to the anatase phase and convert to the rutile phase after being annealed at 900 °C. The obtained highest refractive index of anatase TiO_2_ coating was 2.26. This value increased to 2.57 after further annealing, which can be explained by the development of the crystalline structure, the difference of surface morphology and the increase of the density of the coatings as indicated by AFM and Raman results.
